# Thyroid Homeostasis: An Intricate Network of Production, Transport, Metabolism and Receptors Interaction

**DOI:** 10.3390/ijms23126751

**Published:** 2022-06-17

**Authors:** Annunziatina Laurino, Laura Raimondi

**Affiliations:** Department of Neuroscience, Psychology, Drug Sciences and Child Health (NEUROFARBA), Section of Pharmacology, University of Florence, 50139 Florence, Italy; laura.raimondi@unifi.it

Correct thyroid function is regarded essential for maintaining the growth, differentiation and survival of most mammalian cells at homeostatic conditions. Thyroid function is sustained by self-controlling steps, including the hypothalamus, thyroid hormone production and secretion, its endocrine signaling in target cells’ genomic and non-genomic pathways.

In line with this, perturbing conditions of thyroid function, i.e., hypo and hyperthyroidism, at clinical and subclinical levels, are casually linked to abnormal cell physiology and changes in their phenotype. Despite the increase in knowledge, much remains to be explored about the mechanisms underpinning the loss of thyroid homeostasis and their possible impact on biological systems.

This Special Issue collected papers that explore the novel molecular pathways governing thyroid hormone (TH) biosynthesis and signaling and genetic mutations on thyroid hormone receptors (THRs) as possible causes of the clinical manifestations of thyrotoxicosis, hypo- and hyperthyroidism. Furthermore, this collection indicates a possible role for TH-related compounds as pro-oxidant/antioxidant tissue regulators.

TH secretion is controlled by the hypothalamus-pituitary-axis, that ultimately, through the action of cysteine cathepsins, allows the solubilization of thyroglobulin (Tg) and the secretion of TH into the circulation involving specific transmembrane transporters, including the monocarboxylate transporters type 8 and 10 (Mct8 and Mct10, respectively). Venouglapan et al. [1] demonstrated that mice lacking cathepsin K and TH transporters (Ctsk-/-/Mct8-/y/Mct10-/-) have, as expected, compromised TH transport, showed increased Tg degradation and present self-induced thyrotoxicity. Intra-thyroid TH accumulation, likely caused by ROS-induced stress and/or by increasing cytosolic Ca2+ levels, leads to autophagy and upregulation of the expression of endo-lysosomal proteins, independently from the classic pathway of thyroid auto-regulation. Considering the limitations of the experimental model, this paper highlights the extreme plasticity of the gland and indicates a possible cause of thyrotoxicosis.

Most of the TH effects are mediated by the interaction with two types of THRs, isoform A (THRA) and B (THRB), encoded by two different genes. In turn, two isoforms of THRA, i.e., THRA1 and THRA2, derived from the alternative splicing of THRA mRNA, are described. Several variants of THRA-associated thyroid diseases have been found. Most of these are located in the ligand binding domain of the carboxyl terminal portion of THRA1, causing a receptor loss of function, i.e., inability to bind the active form of TH, triiodothyronine (T3). Paisdzior et al. [2] described a new variant of THRA located between the DNA and the ligand binding domain, thus affecting both THRA1 and THRA2. Interestingly, patients carrying this variant experience symptoms of hypo- and hyper-thyroidism. The authors demonstrated that this variant represents a gain-of-function mutation of THRA1, thus explaining the hyperthyroid symptoms. At the same time, this condition is associated with increasing THRA2 inhibitory effects, possibly predicting the hypothyroid symptoms. Overall, the results suggest that receptors can reciprocally govern their functions.

The subcellular localization of THRs is crucial for thyroid function. Hublein et al. [3] investigated whether THRbeta and THRbeta-1 prevalence and subcellular distribution could be predictive of overall survival (OS) in ovarian cancer patients. These authors demonstrated that the expression of THRbeta was found to be closely related with a shortened OS. In particular, reduced OS was associated with the cytoplasmic localization of THRbeta. This localization, likely exerting non-genomic activity on PI3K and mTOR signaling, might explain ovarian cancer aggressiveness.

In a model of rat embryonic stem cell line, Fernandez et al. [4] unveiled the role of TH at early development during the pre-implantation stage, demonstrating the role of THR and retinoic acid (RA) in neurectoderm maturation. The authors described, for the first time, the expression of THRs, the three isoforms of deiodinases, Mct8 and Mct10, throughout in vitro differentiation until the embryoid body stage. Furthermore, they demonstrated that cell treatment with RA resulted in an enhanced specification of the neuroectodermal lineage, an effect which did not occur when an antagonist of the THR was used. Accordingly, TH binding at THR was a requisite to allow RA-mediated neuroectodermal differentiation.

The impairment of TH homeostasis unbalances the vasoconstriction/dilatation factor levels that impact on the vascular tone. Among the symptoms of hyperthyroidism is enhanced systolic blood pressure, even if vascular resistance is reduced. In an experimental model of hyperthyroidism, [5] Bunsò et al. showed, for the first time, a role for the perivascular innervation in the determination of the vascular resistance of the hyperthyroid subjects. In particular, they found that electrical field stimulation (EFS)-induced vasoconstriction of the mesenteric artery from hyperthyroid rats presented lower adrenergic and enhanced nitrergic and adenosine components in the controls’ arteries.

TH-related compounds are a series of molecules that circulate in mammals, exerting effects somehow related with those of TH by mechanisms largely unknown. Gencarelli et al. [6] investigated the redox properties of two TH-related compounds, the 3-iodothyronamine and 3-iodothyroacetic acid (T1AM and TA1, respectively), at chemical settings and in a model of differentiating rat adipocytes. They found that T1AM and TA1, although with minor differences, work as antioxidants and scavengers when exposed to strong pro-oxidant conditions; instead, in the cell model, T1AM and TA1 mediate pro- or anti-oxidant effects, respectively, probably through the inhibition/activation of sirtuin1 and then exerting post-translational control on protein activities. This work demonstrated for the first time a novel mechanism of action for T1AM and TA1, which could predict some clinical effectiveness.

By exploring the novel aspects of the intricate network relating TH and its target cells, the researches collected in this Special Issue contribute to the understanding of the molecular and genetic basis of thyroid diseases, the role of TH in cell differentiation and of TH function in malignancy.
